# Postpartum Intrauterine Device Removal and Access to Removal in the 18 Months Following an Intervention in Tanzania, Sri Lanka, and Nepal

**DOI:** 10.1111/sifp.70038

**Published:** 2025-10-23

**Authors:** Brooke W. Bullington, Katherine Tumlinson, Leigh Senderowicz, Joanna Maselko, Kavita Shah Arora, Jessie K. Edwards, Audrey Pettifor

## Abstract

Family planning programs in the Global South promote long‐acting reversible contraception (LARC), but research suggests that women face barriers to LARC discontinuation, inhibiting their reproductive autonomy. Scholars have called for improved data visibility around LARC removal access. We use data from the Postpartum Intrauterine Device (PPIUD) Study, a randomized trial of a PPIUD intervention conducted in Nepal, Sri Lanka, and Tanzania from 2015 to 2018. Among women who adopted PPIUDs, we describe PPIUD status (in‐use, expelled, deliberately removed) at three follow‐up points. We report the proportion of participants who sought PPIUD removal and, among those, whether they faced barriers to removal. About three‐quarters of 5370 participants had their PPIUD in use 18 months following insertion; one‐fifth had their PPIUD deliberately removed, and 6 percent had their PPIUD expelled. Of the 22 percent of participants who sought PPIUD removal, a quarter faced a barrier to removal; most barriers were provider‐imposed. In conjunction with existing literature, our findings highlight that barriers to LARC removal are structural, rooted in larger public health and demographic goals that aim to promote contraceptive uptake. We call for safeguards to ensure that people who desire LARC removal can discontinue their method.

## INTRODUCTION

Long‐acting reversible contraception (LARC), which includes subdermal implants and intrauterine devices (IUDs), has been lauded by researchers and clinicians as “first line” due to its safety, effectiveness, easy maintenance, and long duration of use (Curtis and Peipert 2017; Stoddard, McNicholas, and Peipert 2011; Secura et al. 2010). Because LARC has been considered underutilized in low‐ and middle‐income countries (LMICs), family planning programs have increasingly promoted these methods in recent decades (Braun and Grever 2020; Jacobstein 2018; Cleland et al. 2017). Programs that expand access to the implant, like the donor‐funded Implant Access Initiative, have been deemed successful by program evaluators; there has been a substantial increase in the prevalence of implant use in LMICs (Jacobstein 2018; Braun and Grever 2020). In fact, in some LMICs, including Burkina Faso and Kenya, the implant has become one of the most commonly used contraceptive methods (Tumlinson et al. 2023). Uptake of IUDs, on the other hand, has remained what international funding agencies and researchers have deemed “low” (United Nations Department of Economic Affairs, and Division 2020; Ali, Folz, and Farron 2019; Cleland et al. 2017). In the past 10 years, government foundations and large international organizations have therefore funded initiatives to “revitalize the IUD agenda” and increase IUD prevalence, with a particular focus on the postpartum period to increase interpregnancy intervals (Brunie et al. 2021; Cleland et al. 2017; Danna et al. 2022; Harrison and Goldenberg 2017).

Increasing the availability of a wide array of contraceptive methods, including IUDs, is imperative to promote autonomy in contraceptive decision‐making. In fact, full choice—that is, whether an individual can make a decision about contraception with access to a wide range of methods—is one of the three subdomains of Senderowicz's conception of contraceptive autonomy (Senderowicz 2020). Studies have shown that individuals have unique and diverse preferences regarding contraceptive attributes (Yeh et al. 2022); increasing the number of methods available can help ensure that these preferences can be realized. Thus, expanding access to LARC is an important step toward person‐centered family planning service delivery.

However, evidence suggests that programs to introduce or expand access to certain contraceptive methods in LMICs have, at times, inhibited access to other methods and thereby devalued individual preferences. For example, studies evaluating a postpartum intrauterine device (PPIUD) intervention in Tanzania found that the intervention led providers to emphasize the IUD and de‐emphasize other methods in their contraceptive counseling (Senderowicz et al. 2021, 2023). This type of method overpromotion may be especially common among programs that are evaluated primarily based on uptake‐focused measures of success—that is, whether people adopted the PPIUD—rather than measures of quality of care, agency, or autonomy (Senderowicz 2020; Speizer, Bremner, and Farid 2022; Holt et al. 2024). Overpromotion may also be common when the methods being introduced are situated as “better” than other methods available. This can be particularly concerning in the context of medical paternalism, when well‐meaning providers believe they know what contraceptive option suits a person better than that person themselves, or providers insert their own beliefs about who should or should not be contracepting or bearing children (Brandi and Fuentes 2020).

Reproductive justice scholars have long cautioned against the “LARC‐first” approach to contraceptive programming and counseling, in which long‐acting methods are emphasized and promoted as the “best” contraceptive option for most or all users, while short‐acting methods are de‐emphasized or ignored (Brandi and Fuentes 2020; Gomez, Fuentes, and Allina 2014; Gubrium et al. 2016; Higgins 2014; Mann 2022). As Gubrium and colleagues state, “positioning any method as the first‐line choice invites a lack of regard for the preferences of people who have the capacity to get pregnant” (Gubrium et al. 2016). Scholars have further argued that framing LARC as a solution to social problems, a panacea for unintended pregnancy, or a means for reducing population growth ignores and potentially contributes to deeply rooted structural inequities and the history of eugenics and reproductive coercion that has targeted low‐income women, women of color, and women from the Global South (Gubrium et al. 2016; Higgins 2014; Brandi and Fuentes 2020; Gomez, Fuentes, and Allina 2014; Takeshita 2011). This framing places the onus of these structural problems on individual reproductive choices and puts pressure on providers to promote certain methods as a means of “transforming statistics” (Gubrium et al. 2016; Brandi and Fuentes 2020).

Another concern of the “LARC‐first” approach is that LARC is what Clarke called “imposable,” that is, controlled by clinicians and not the method users themselves (Clarke 2000). Individuals wishing to adopt or discontinue LARC rely on a trained provider for insertion and removal. Scholars have warned that the “imposability” of LARC makes it “inherently more capable of being used coercively” (Brandi and Fuentes 2020). While a small body of literature explores IUD self‐removal, this means of users regaining control of discontinuation has not been widely studied or promoted (Foster et al. 2014; Amico et al. 2020; Broussard and Becker 2021; Cartwright et al. 2022).

In the attempt to understand the impacts of LARC “imposability” on reproductive autonomy, it is imperative to consider the history of the IUD's development. The “imposability” of the IUD was an intentional decision in the method's initial design. As Takeshita outlines in *The Global Biopolitics of the IUD*, IUD researchers developed the method in response to widespread fear of overpopulation in the Global South, in search of a “method for the masses” that required less “motivation” from users (Takeshita 2011). Part of the goal was, therefore, to develop a method that could be imposed upon people and not easily discontinued (Takeshita 2011). Writing of the benefits of the IUD, Alan Guttmacher, president of the Planned Parenthood Federation, said, “No contraceptive could be cheaper, and also, once the damn thing is in, the patient cannot change her mind” (Takeshita 2011).

Evidence suggests that people who desire to discontinue LARC have faced barriers to removal since the methods’ inception. In clinical trials for Norplant, the first subdermal contraceptive implant, providers refused to remove the implant even after repeated requests in Egypt, Bangladesh, and Indonesia (Hardee et al. 1994; Morsy 1995; Hardee, Balogh, and Villinskp 1997). A 1990 study of women in the clinical trial in Bangladesh found that, of women who requested implant removal, nearly a quarter were not able to achieve removal at the facility where their implant was inserted, and 15 percent still had their implant in place despite desire for removal (Hardee et al. 1994). Barriers to discontinuation were particularly prominent among women who requested removal before the five‐year method expiration (Hardee, Balogh, and Villinskp 1997). Though program implementers collected and reported data on barriers to removal in these trials, such barriers continued to persist even after product introduction (Pleaner et al. 2017).

A growing body of evidence has highlighted the numerous barriers people face when seeking LARC removal in Africa, Europe, North America, and Australia. Though these settings vary drastically from a social, economic, and political perspective, similar themes in barriers to LARC removal emerge across studies. Nearly every study examining barriers to LARC removal has identified provider reluctance or refusal to remove methods “early”—prior to method expiration—as one of the primary reasons people cannot achieve removal (Ding et al. 2021; Lebetkin et al. 2022; Amico et al. 2016, 2017; Caddy et al. 2022; Utaile et al. 2020; Yirgu et al. 2020; Senderowicz 2019; Senderowicz and Kolenda 2022; Manzer and Bell 2022; Callahan et al. 2020; Mann et al. 2019; Britton et al. 2021; Higgins, Kramer, and Ryder 2016; Costenbader et al. 2020; Hoggart, Louise Newton, and Dickson 2013; Howett et al. 2021; Brunie et al. 2022; Wollum et al. 2024; Ela et al. 2022; Fox et al. 2024). In a qualitative study in an anonymized sub‐Saharan African country, Senderowicz and Kolenda describe that providers view reasons for “early” LARC removal as either “legitimate” or “illegitimate,” with illegitimate reasons including desire for removal, side effects, and desire for pregnancy (Senderowicz and Kolenda 2022). Patients, therefore, can feel as though they need to make a “convincing case” to obtain removal, and that desire for discontinuation is often insufficient (Mann et al. 2019). Studies have further documented that providers use persuasion and delay tactics, such as treating the side effects of the method rather than removing the method altogether, to avoid removal (Britton et al. 2021; Manzer and Bell 2022; Amico et al. 2016; Senderowicz and Kolenda 2022; Callahan et al. 2020; Ding et al. 2021; Amico et al. 2017). Further documented barriers to removal include cost (Romero et al. 2020; Britton et al. 2021; Callahan et al. 2020; Mann et al. 2019), lack of provider training (Lebetkin et al. 2022; Cartwright et al. 2023), lack of supplies (Senderowicz et al. 2022; Lebetkin et al. 2022; Cartwright et al. 2023), and lack of access to a facility equipped to remove LARC (Cartwright et al. 2023).

Barriers to desired LARC removal violate medical guidelines, human rights laws, and bodily autonomy, leaving people unable to make decisions about their reproductive lives. Further, they contribute to medical mistrust and poor patient–provider relationships (Caddy et al. 2022). Given the importance of expanding the contraceptive method mix globally, the continued promotion of LARC (and specifically the IUD) in LMICs, and the increased prevalence of their use, scholars have urgently called for additional research on LARC removal, including improved measurement and data visibility (Christofield and Lacoste 2016; Howett et al. 2019). Most studies that examine LARC removal in LMICs focus on the implant, given its high prevalence; more research is needed to understand barriers to IUD removal. Further, no studies that examine access to IUD removal have focused on the postpartum IUD. Given that many IUD interventions focus on the postpartum period (Harrison and Goldenberg 2017; Cleland et al. 2017; Pleah et al. 2016), understanding postpartum IUD removal is important. Finally, as many studies that explore barriers to LARC removal are qualitative, there is a need for research that quantifies the prevalence of such barriers and explores their correlates.

An array of studies have demonstrated that individual‐level characteristics, such as a woman's age and parity, influence contraceptive counseling and access (Tumlinson, Okigbo, and Speizer 2015; Tumlinson et al. 2013). For example, studies show that those who are unmarried or have few children are often denied family planning services (Tumlinson, Okigbo, and Speizer 2015; Tumlinson et al. 2013). A study in Burkina Faso found that 16 percent of women with children report they have ever been “encouraged” to use family planning because they have “too many” children (Bullington et al. 2023). Because these factors influence contraceptive provision, they may also influence accessibility of LARC removal services, though this has been scantly explored.

To fill these gaps, this study aims to describe 18‐month trends in IUD removal and access to removal following a randomized PPIUD intervention implemented in Nepal, Sri Lanka, and Tanzania. We quantify barriers to LARC removal over time and explore whether demographic characteristics are associated with experiencing barriers to removal using longitudinal data.

## METHODS

### Background

This analysis uses data from the PPIUD Study, a step‐wedged, cluster‐randomized trial of a PPIUD intervention that was conducted in Nepal, Sri Lanka, and Tanzania from 2015 to 2018.

The intervention was designed by the International Federation of Gynaecology and Obstetrics (FIGO) and implemented by in‐country partners. The study has been described in depth previously (Canning et al. 2016). In brief, the intervention involved training of clinicians through classroom‐based sessions and on‐the‐job training and mentoring. It also included training on postpartum contraceptive counseling for midwives, skilled birth attendants, and community health workers. Women presenting at hospitals with the PPIUD intervention received information on postpartum contraception and the availability of PPIUD services, including PPIUD counseling and exposure to leaflets and videos about PPIUDs.

In each country, six hospitals were selected based on geographic diversity and matched into pairs by annual number of deliveries. One hospital in each pair was randomly assigned to the intervention group. The randomly selected facilities received the hospital standard‐of‐contraceptive care for the first three months of the study and the PPIUD intervention period for the next nine (Tanzania) or 15 months (Nepal and Sri Lanka), while the other facility in each country received the standard‐of‐care for first nine months and the PPIUD intervention for the subsequent three (Tanzania) or nine months (Nepal and Sri Lanka).

The PPIUD Study enrolled women who delivered a baby at any of the selected hospitals during the study period. In Tanzania, women 18 years of age and older were eligible, whereas in Nepal and Sri Lanka, there was no lower age limit for enrollment. Women completed a baseline survey and up to three follow‐up surveys at four to eight weeks postpartum, 9–12 months postpartum, and 18–24 months postpartum.

Participants were not compensated for study participation or PPIUD uptake. Ethical approval for this study was provided by the Ethics Review Committee at the Faculty of Medicine at the University of Colombo in Sri Lanka, the Nepal Health Research Council, and the National Institute for Medical Research in Tanzania. The Institutional Review Boards at Harvard University and the University of North Carolina at Chapel Hill deemed this study exempt.

### Data Collection and Analytic Sample

The study team developed surveys in English, which were then translated into local languages. Surveys were pretested in each country and modified based on feedback. The CommCare data collection app was used to collect all data on Android tablets.

Trained female enumerators administered the baseline survey to eligible women in postnatal hospital wards after delivery and immediate PPIUD insertion (if applicable) but prior to hospital discharge. Baseline surveys contained questions about demographic characteristics, contraceptive counseling, PPIUD‐specific counseling, and PPIUD insertion. The baseline questionnaire also contained a question for providers to confirm immediate postpartum IUD insertion if a woman reported receiving a PPIUD. In this analysis, only women who had a confirmed immediate PPIUD insertion were included, as our primary outcome was IUD removal.

Women who received the PPIUD were invited for a two‐month follow‐up visit at the study hospitals. Women who were not available for an in‐person interview completed their two‐month follow‐up visit via phone (14 percent of participants who received a PPIUD in Nepal, 41 percent in Sri Lanka, 18 percent in Tanzania). The two‐month follow‐up survey contained questions about experiences and satisfaction with the PPIUD, including whether the PPIUD had been deliberately removed or expelled. Women who attended the two‐month visit in person underwent a medical exam to confirm the status of the PPIUD. Women who attended the two‐month visit via phone were asked about the results of their PPIUD exam if it had been previously conducted, and if not, were asked about whether their PPIUD had been deliberately removed or expelled.

Women also completed follow‐up surveys 9 and 18 months postpartum in person at study hospitals, their homes, or satellite clinics. During follow‐up interviews, women were asked similar questions about their experiences with the PPIUD and barriers they faced when seeking PPIUD removal. No medical examinations were conducted at 9‐ and 18‐month follow‐up.

### MEASURES

#### Participant Characteristics

Demographic characteristics were collected during baseline surveys. Age in years was measured continuously and categorized as < 21, 21–25, 26–30, 31–35, >35. Partnership status was captured as binary (married or living with partner/not married or living with partner). Parity was measured continuously and subsequently categorized as 1, 2, 3, 4, 5, or >5. Education level was categorized as none, some primary school, some secondary school, completed secondary school, or more than secondary school.

#### PPIUD Status (In‐Use versus Deliberately Removed or Expelled)

The status of the PPIUD was determined at each follow‐up visit. At two‐month follow‐up, participants were classified as having their PPIUD deliberately removed if they either (1) reported that their PPIUD had been deliberately removed at their in‐person or phone visit or (2) had their PPIUD deliberately removed during their in‐person visit. Participants were classified as having their PPIUD expelled if they either (1) reported that their PPIUD had not been deliberately removed but did not have an IUD present in the medical examination at their in‐person visit, (2) reported that they had attended a check‐up for their PPIUD and were told that their PPIUD was not present and in place, or (3) reported that they had not attended a check‐up for their PPIUD but thought that their PPIUD was not present and in place. Participants for whom status of PPIUD could not be determined (7 percent in Nepal, 3 percent in Sri Lanka, 11 percent in Tanzania) were categorized as having their IUD in‐use at two‐month follow‐up to ensure that estimates of expulsion or removal were conservative.

At 9‐ and 18‐month follow‐up, PPIUD removal was determined based on whether a participant responded “Yes” to, “At any point after you received the PPIUD, was the PPIUD deliberately removed at your request?” PPIUD expulsion was determined based on whether a participant responded “Yes” to, “At any point after you received the PPIUD, was the PPIUD expelled (fell out without being intentionally removed)?” If a participant reported that their PPIUD had been deliberately removed or expelled at any visit, they were classified as having their PPIUD deliberately removed or expelled at all subsequent visits. Participants who reported that their PPIUD had been both deliberately removed and expelled were categorized as having their PPIUD deliberately removed and not expelled.

#### Attempted Removal and Removal Barriers

We assessed attempted PPIUD removal at 9‐ and 18‐month follow‐up visits but not two‐month follow up because questions were not asked. We categorized participants as having sought PPIUD removal if they (1) reported their PPIUD had been deliberately removed or (2) answered “Yes” to, “At any point since you received the PPIUD, have you sought PPIUD removal services?” Once a participant reported they sought removal at a given visit, they were categorized as having sought removal at subsequent visits.

We determined whether a participant faced a barrier to PPIUD removal at 9‐ and 18‐month follow‐up. We categorized participants as having faced any barrier to removal if they (1) reported that their PPIUD had been deliberately removed and answered “Yes” to, “At any point, did a health service provider refuse to remove the PPIUD when you requested removal?” or (2) reported that they had sought PPIUD removal but did not report that their PPIUD had been deliberately removed. Once a participant reported they faced a barrier to removal, they were categorized as having faced a barrier to removal at subsequent visits.

We also determined whether participants who sought removal faced a provider‐imposed barrier to removal at 9‐ and 18‐month follow‐up. We categorized participants as having experienced a provider‐imposed barrier to removal if they answered “Yes,” “At any point, did a health service provider refuse to remove the PPIUD when you requested removal?,” regardless of whether their PPIUD had been removed. Once a participant reported they faced a provider‐imposed barrier to removal, they were categorized as having faced a provider‐imposed barrier to removal at subsequent visits. Those who reported that a provider had refused to remove their PPIUD were asked how many health providers they had requested removal from and reasons for the provider's refusal using predefined, select‐all‐that‐apply response categories.

### Missing Data

Visits were considered missing if a participant either (1) did not complete the visit or (2) completed the visit but did not answer questions about PPIUD removal or expulsion. In Nepal, 125 participants (8 percent) were missing two‐month follow‐up, 207 (13 percent) were missing nine‐month follow‐up, and 202 (13 percent) were missing 18‐month follow‐up. In Sri Lanka, two participants (0 percent) were missing two‐month follow‐up, 514 (18 percent) were missing nine‐month follow‐up, and 766 (28 percent) were missing 18‐month follow‐up. In Tanzania, 204 participants (19 percent) were missing two‐month follow‐up, 486 (45 percent) were missing nine‐month follow‐up, and 351 (33 percent) were missing 18‐month follow‐up. In Tanzania, both 9‐ and 18‐month follow‐ups coincided with the measles vaccination schedule. Follow‐up at 18 months was more intensive, with research assistants contacting participants to encourage attendance, likely contributing to lower loss‐to‐follow‐up compared to nine months.

To account for attrition, we created inverse probability of observation weights. We used causal diagrams to identify baseline demographic and facility‐level factors needed for d‐separation between missingness and key outcomes, attempted IUD removal, and IUD removal in each country. Variables included age, education level, parity, region, contraceptive use, and study hospital. In Tanzania, these variables were missing for *n* = 45 participants (4 percent of PPIUD users in the Tanzania sample); these participants were excluded from analysis. We used logistic regression to estimate the probability of attending a given follow‐up visit conditional on these variables at each time point. The weight for participants who completed a given follow‐up visit was the inverse probability of attending that visit, conditional on the covariates listed above. Separate weights were developed for each country at each follow‐up visit.

### Statistical Analysis

We used descriptive statistics to report participant characteristics. We described the number and proportion of participants with each PPIUD status (in‐use, expelled, deliberately removed) at each follow‐up visit. At 9‐ and 18‐month follow‐up, we also described the proportion of participants who reported seeking PPIUD removal and, among those who did seek removal, the proportion of participants who faced any barrier to PPIUD removal, reported a provider‐imposed barrier to PPIUD removal, had their PPIUD removed, and did not have their PPIUD removed. Among participants who reported a provider‐imposed barrier to removal, we reported the number providers approached for removal and the reason for refusal. We reported statistics disaggregated by country and for the total sample.

For each country at 18‐month follow‐up, we calculated the probability that participants fell into four exclusive groups (1) PPIUD expelled before seeking removal, (2) did not seek removal, (3) sought PPIUD removal and experienced barriers, and (4) sought PPIUD removal and did not experience barriers (Online Figure A1). We did this by dividing the number of participants who fell into a given category by the total number of participants at 18‐month follow‐up. We then calculated the probability that a participant was in each of the above categories by baseline sociodemographic characteristics, including parity, age, and education level. These characteristics were determined a priori, as previous literature has shown that they are associated with bias in contraceptive counseling (Tumlinson, Okigbo, and Speizer 2015; Tumlinson et al. 2013). We calculated the probability of being in each category for different levels of each characteristic (i.e., for parity: probability of being in each category among those with parity 1 and those with parity 2+). We then calculated differences and 95 percent confidence intervals for the probability of seeking removal and experiencing barriers for each level of baseline characteristics compared to a referent group determined a priori. We examined probabilities and probability differences separately by country, as factors associated with barriers to removal may differ.

## RESULTS

### Participant Characteristics

**FIGURE 1:**
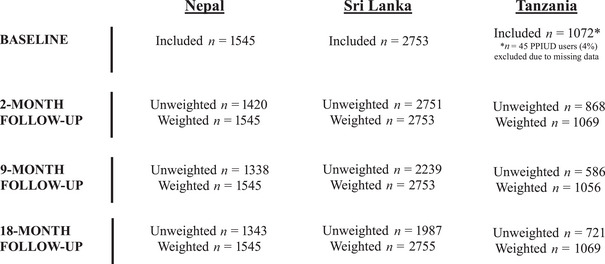
Flow chart of included PPIUD users at baseline and follow‐up in Nepal, Sri Lanka, and Tanzania

In total, 5370 women adopted postpartum IUDs and were included in this analysis (1545 from Nepal, 2753 from Sri Lanka, and 1072 from Tanzania; Figure 1 and Table 1). In the total sample, median age was 27 (interquartile range [IQR]: 23, 31); most participants were 21–30 years old. Nearly all included women were married (99 percent). Median parity in the total sample was 2 (IQR: 1, 2). More than half of the sample had completed secondary school.

**TABLE 1:** Baseline characteristics of women who adopted intrauterine devices immediately postpartum in Nepal, Sri Lanka, and Tanzania

	Nepal		Sri Lanka		Tanzania		Total	
	(*n* = 1545)		(*n* = 2753)		(*n* = 1072)		(*n* = 5370)	
	*n*	Percentage	*n*	Percentage	*n*	Percentage	*n*	Percentage
**Age (years)**								
<21	291	19%	253	9%	143	13%	687	13%
21–25	598	39%	732	27%	238	22%	1568	29%
26–30	438	28%	953	35%	298	28%	1689	31%
31–35	162	10%	608	22%	233	22%	1003	19%
>35	56	4%	207	8%	160	15%	423	8%
Median [interquartile range]	25	[22,28]	28	[24,31]	28	[23,33]	27	[23,31]
**Married or living with partner**	1545	100%	2748	100%	1033	96%	5326	99%
**Parity**								
1	451	29%	1044	38%	332	31%	1827	34%
2	771	50%	1272	46%	250	23%	2293	43%
3	225	15%	378	14%	203	19%	806	15%
4	61	4%	47	2%	138	13%	246	5%
5	25	2%	11	0%	84	8%	120	2%
>5	12	1%	1	0%	65	6%	78	1%
Median [interquartile range]	2	[1,2]		2 [1,2]	2	[1,4]	2	2 [1,2]
**Education**								
None	180	12%	34	1%	8	1%	222	4%
Some primary	131	8%	69	3%	64	6%	264	5%
Completed primary	101	7%	69	3%	555	52%	725	14%
Some secondary	487	32%	424	15%	57	5%	968	18%
Completed secondary	238	15%	735	27%	261	24%	1234	23%
More than secondary	408	26%	1422	52%	127	12%	1957	36%

### PPIUD Status and Percent Seeking Removal Services

**FIGURE 2:**
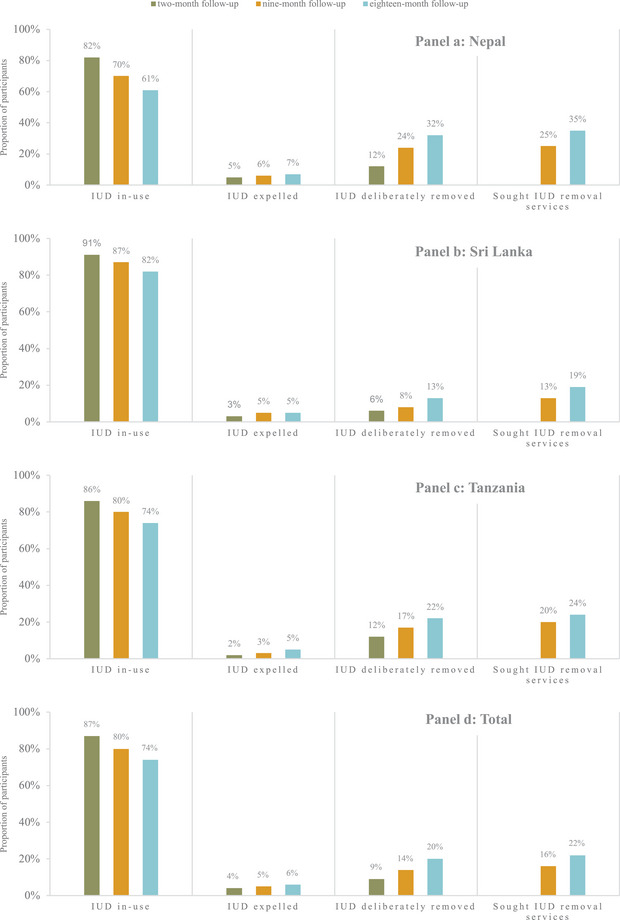
Proportion of PPIUD users who had their IUD in use, had sought IUD removal services^a^, had their IUD expelled, or had their IUD deliberately removed at 2‐, 9‐, and 18‐month follow‐up by country and overall NOTE: ^a^Participants were not asked if they had sought IUD removal services at two‐month follow‐up.

We present the proportion of participants who had their PPIUD in use, expelled, and deliberately removed at each follow‐up visit in Figure 2 and Online Table A1. In all countries, the proportion of participants who had their IUD in use decreased over time, and the proportion of participants who had their PPIUD expelled or deliberately removed increased. In the total sample at 18‐month follow‐up, 74 percent had their PPIUD in use, 6 percent had their PPIUD expelled, and 20 percent had their PPIUD deliberately removed. A higher proportion of participants in Sri Lanka had their PPIUD in use at 18‐month follow‐up (82 percent) compared to Nepal (61 percent) and Tanzania (74 percent), and a lower proportion of participants in Sri Lanka had their PPIUD deliberately removed (13 percent) compared to Nepal (32 percent) and Tanzania (22 percent).

We present the proportion of participants who reported having sought PPIUD removal services at 9‐ and 18‐month follow‐up in Figure 2 and Online Table A1. The proportion of participants who sought removal increased over time in all countries. In the total sample at 18‐month follow‐up, 22 percent of participants reported having sought removal services. The proportion of participants who reported having sought removal services was highest in Nepal (35 percent), followed by Tanzania (24 percent) and Sri Lanka (19 percent).

### Barriers to PPIUD Removal

**FIGURE 3:**
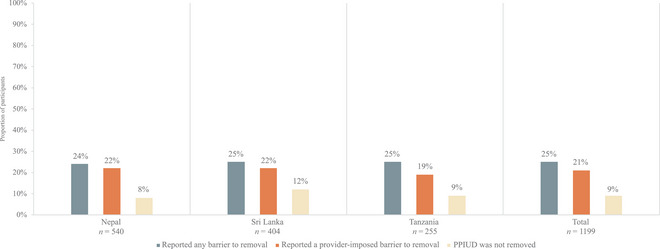
Proportion of PPIUD users who reported barriers to removal and did not achieve removal among those who sought IUD removal at 18‐month follow‐up

We present barriers to removal and removal outcomes among those who sought removal in Figure 3 and Online Table A2. Across all countries, the proportion of participants who reported any barrier to PPIUD removal and provider‐imposed barriers to removal increased over time. In the total sample of participants who sought removal by 18‐month follow‐up (*n* = 1199, 22 percent of the total sample), a quarter of participants (25 percent) reported that they faced any barrier to IUD removal, and 21 percent of participants reported that they faced a provider‐imposed barrier to removal. Further, 9 percent of participants who sought IUD removal did not have their IUD removed. The proportion of participants who sought removal but did not have their IUD removed was highest in Sri Lanka (12 percent), followed by Tanzania (9 percent) and Nepal (8 percent).

In Table 2, we present participant‐reported number of providers approached for PPIUD removal and reasons for provider refusal among those who reported a provider‐imposed barrier to IUD removal at 18‐month follow‐up. Of participants who reported a provider‐imposed barrier to removal (21 percent of participants who sought removal), over a quarter reported approaching more than one provider for removal (27 percent in Nepal, 35 percent in Sri Lanka, and 17 percent in Tanzania). In Nepal and Sri Lanka, the most common reason for provider refusal was that the participant was encouraged to use the IUD for longer (57 percent and 78 percent, respectively). This was the second most common reason for refusal in Tanzania (41 percent). In Tanzania, the most common reason was that the participants were told side effects would go away (71 percent); 26 percent and 23 percent listed this reason in Nepal and Sri Lanka, respectively. In Nepal, 11 percent of participants who faced provider refusal reported that the provider told them that they should not have more children, and 8 percent were told they should not waste the IUD.

**TABLE 2:** Participant‐reported number of providers approached for intrauterine device removal and reasons for provider refusal to remove, among postpartum intrauterine device adopters who reported that a provider had refused to remove their PPIUD, 18‐month follow‐up

	Nepal	Sri Lanka	Tanzania
	(*n* = 120)	(*n* = 88)	(*n* = 49)
Number of providers approached for removal			
1	73%	65%	75%
2	21%	21%	12%
3	5%	12%	5%
4	1%	0%	0%
5	0%	2%	0%
Unknown	0%	0%	7%
Participant‐reported reason for provider refusal^a^			
Encouraged to try longer	57%	78%	41%
Provider said side effects would go away	26%	23%	71%
Provider said woman should not have more children	11%	0%	0%
Provider said woman should not waste IUD	8%	2%	3%
Other	24%	12%	29%
Don't know	4%	2%	0%

^a^Participants could select multiple reasons for provider refusal to remove their IUD.

### Barriers to Removal by Characteristics (Parity, Age, Education)

**FIGURE 4:**
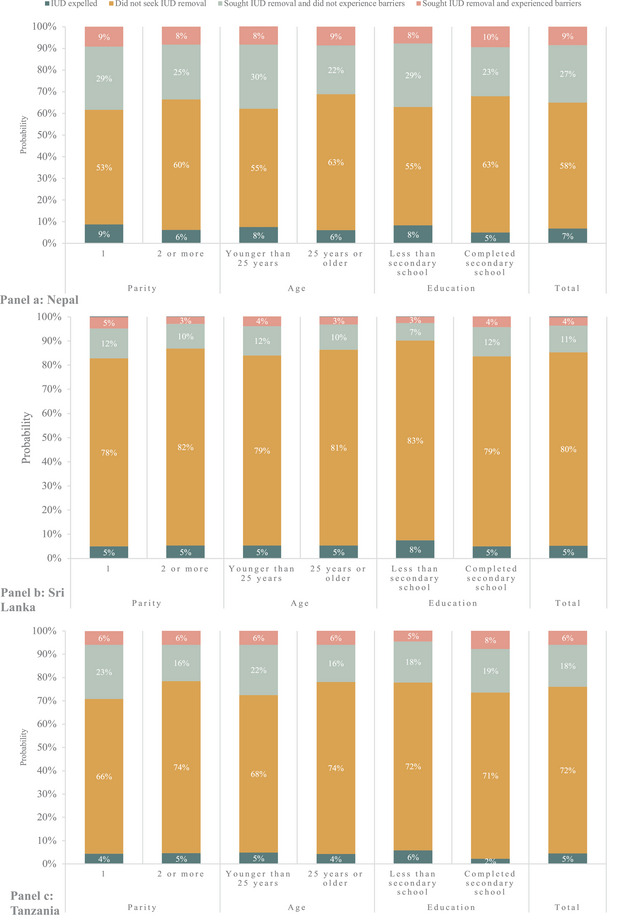
Probability that PPIUD users had their IUD expelled, did not seek IUD removal, sought IUD removal and did not experience barriers, and sought removal and experienced barriers by baseline characteristics (age, parity, education)

We report the probability that participants had their IUD expelled, did not seek IUD removal, sought removal and did not experience barriers, and sought removal and experienced barriers at 18‐month follow‐up overall and by baseline demographic characteristics in Figure 4 and Online Table A3. Most participants did not seek IUD removal (58 percent in Nepal, 80 percent in Sri Lanka, 72 percent in Tanzania). In all countries, the probability of not seeking removal was higher among those with parity of 2 or more (compared to parity of 1) and age 25 years or older (compared to less than 25 years). In Nepal, participants who had completed secondary school had a higher probability of not seeking removal compared to those who had not completed secondary school. In our total sample, the probability of having sought IUD removal and not experiencing barriers ranged from 11 percent (Sri Lanka) to 27 percent (Nepal).

In Table 3, we present differences in the probability of having sought IUD removal and experiencing barriers by parity, age, and education. In all countries, there were no statistically significant associations between age and the probability of having sought removal and experiencing barriers. In Tanzania and Nepal, there were no statistically significant associations between parity and having sought removal and experiencing barriers. In Sri Lanka, participants with two or more children had a significantly lower probability of having sought removal and experiencing barriers than those who had one child (difference in probability: −1.8 percent; 95 percent confidence interval: −3.3 percent, −0.3 percent). In Nepal and Sri Lanka, there were no statistically significant associations between education level and having sought removal and experiencing barriers. In Tanzania, participants who had not completed secondary school had a significantly lower probability of having sought removal and experiencing barriers compared to those who had completed secondary school (difference = −3.2 percent; 95 percent confidence interval = −6.3 percent, −0.05 percent).

**TABLE 3:** Differences in the probability of seeking IUD removal and experiencing barriers by baseline demographic characteristics (parity, age, education) at 18‐month follow‐up

	Nepal	Sri Lanka	Tanzania
Parity (births)			
1 (ref)	0	0	0
2 or more	−1.1% (−4.2%, 2.1%)	−1.8% (−3.3%, −0.3%)	−0.04% (−3.1%, 3.0%)
Age (years)			
25 or older (ref)	0	0	0
Younger than 25	−0.4% (−3.2%, 2.4%)	−1.2% (−2.7%, 0.3%)	−0.0% (−3.0%, 2.9%)
Education			
Completed secondary school (ref)	0	0	0
Less than secondary school	−1.9% (−4.7%, 1.0%)	−1.3% (−2.8%, 0.2%)	−3.2% (−6.3%, −0.05%)

## DISCUSSION

In this longitudinal study examining trends in PPIUD removal and access to removal following a PPIUD promotional intervention in Nepal, Sri Lanka, and Tanzania, we found that one‐fifth of participants discontinued PPIUD use by 18‐month follow‐up. Still, PPIUD removal was not accessible for some participants. Of the 22 percent of participants who sought PPIUD removal, a quarter faced barriers to removal. Most barriers were provider‐imposed, with a provider refusing to remove the PPIUD when asked. Nearly one in 10 women who sought PPIUD removal did not have their PPIUD removed during the study period. In this context, individual characteristics were mostly not significantly associated with whether participants faced barriers when seeking removal. In conjunction with existing literature, our findings suggest that barriers to LARC removal may be linked to structural incentive systems in family planning programs that promote contraceptive uptake over reproductive autonomy and may not always result from providers universally targeting certain subgroups of those seeking removal.

We found that 22 percent of participants who adopted PPIUDs sought discontinuation within 18 months, indicating potential method dissatisfaction, contraceptive counseling that did not center on contraceptive preferences, or desire for pregnancy, among other fluid and dynamic reasons for ceasing method use. Normalizing method discontinuation or switching as a neutral outcome for any reason, be it desire for additional children, life changes, side effects, etc., is essential to ensure individuals can have their reproductive needs met. Our findings are in line with prior research that shows 12‐month discontinuation rates of copper IUDs between 3 percent and 27 percent (Costescu et al. 2022). Discontinuation in the PPIUD study could be influenced, in part, by limited counseling, as prior research in Tanzania found that participants who delivered in facilities with the PPIUD intervention were less likely to be counseled on other contraceptive methods (Senderowicz et al. 2021; 2023). Participants may not have received information on an array of contraceptive methods and adopted the IUD even if it did not fit their contraceptive needs or another method would have suited them better. While introducing new contraceptive methods, it is imperative to promote full contraceptive choice and provide participants with information on and access to resources that enable method discontinuation and switching for any reason. Programs that promote a single method must be cautious about potential method overpromotion that inhibits reproductive autonomy. Further, programs should be evaluated based on whether they promote free choice—that is, whether people can decide to use or not use contraception voluntarily without barriers or coercion (Senderowicz 2020).

Our findings reveal the urgent need for family planning programs—particularly those that offer or promote LARC—to provide safe, affordable, and accessible removal services for all people who desire discontinuation. More resources for programs and service providers, including increased funding for personnel, provider training, and supplies, would likely alleviate some barriers. However, one‐fifth of participants in our study who sought PPIUD removal faced a provider‐imposed barrier in which a provider refused to remove their PPIUD upon request. Several additional studies have found provider refusal to remove LARC as one of the most common barriers (Ding et al. 2021; Lebetkin et al. 2022; Amico et al. 2016, 2017; Caddy et al. 2022; Utaile et al. 2020; Yirgu et al. 2020; Senderowicz 2019; Senderowicz and Kolenda 2022; Manzer and Bell 2022; Callahan et al. 2020; Mann et al. 2019; Britton et al. 2021; Higgins, Kramer, and Ryder 2016; Costenbader et al. 2020; Hoggart, Louise Newton, and Dickson 2013; Howett et al. 2021; Brunie et al. 2022; Wollum et al. 2024; Ela et al. 2022; Fox et al. 2024).

In the context of prior research and the history of the IUD's development as an imposable method (Takeshita 2011), our findings align with the idea that provider refusal to remove LARC may not always be related to individual providers or limited to just one program or country. Rather, provider‐imposed barriers to LARC removal exist throughout family planning programming in the Global South, likely linked to the underlying goals and motivations of family planning programs. For example, during the PPIUD intervention, FIGO's central team in London developed and updated a real‐time dashboard to provide feedback to clinicians and in‐country project leaders on numerous metrics, including the number of PPIUD insertions, PPIUD removals, and PPIUD expulsions (de Caestecker et al. 2018). In‐country staff members, including providers, were regularly updated on progress toward meeting the study's goals, which were primarily focused on PPIUD counseling and uptake. Despite broader stated goals of the program of increasing contraceptive access, the narrow focus of monitoring on PPIUD adoption and continuation may have created alternative incentives for program employees and providers, and perhaps contributed to provider refusal to remove PPIUDs to improve statistics (Senderowicz et al. 2023). Recognizing the structural roots of barriers to LARC removal, including the ways that top‐down incentive systems may unintentionally inhibit autonomous contraceptive decision‐making, is critical. These structural factors can often intersect with interpersonal biases related to characteristics like race, class, income, and gender, which can further constrain access. Acknowledging and addressing these overlapping dynamics is necessary to improve service delivery.

Our finding that individual demographic characteristics, like age and parity, were not significantly associated with barriers to PPIUD removal in some settings further highlights the importance of examining barriers to LARC removal as structural, in addition to interpersonal. Though many studies have found that age and parity are associated with provider‐imposed barriers to contraceptive access (Tumlinson, Okigbo, and Speizer 2015; Tumlinson et al. 2013), we find that age was not significantly associated with barriers to PPIUD removal. In Tanzania, those with less education had a significantly lower probability of seeking PPIUD removal and experiencing a barrier compared to those with more education, though this was not the case in Nepal or Sri Lanka. In Sri Lanka, those with two or more children had a significantly lower probability of seeking removal and experiencing a barrier compared to those with one child; this association did not occur in Nepal and Tanzania. These findings reveal that barriers to removal in the PPIUD Study were likely not driven by provider biases against, for example, those who were older or of higher parity. These results mirror previous work in Kenya that found that individual characteristics were not significantly associated with the provider‐imposed contraceptive coercion (Bullington et al. 2024). Together, this evidence suggests that experiences of upward contraceptive coercion—in which people feel pressured to use contraception—can be structural, shaped by program incentive systems. At the same time, further investigating interpersonal biases and their influence on LARC removal in other contexts is important for future research.

The reproductive justice framework, which calls for “the human right to maintain personal bodily autonomy, have children, not have children, and parent the children we have in safe and sustainable communities” through analysis of structural power, is imperative for contextualizing findings related to barriers to LARC removal (Reproductive Justice — Sister Song, n.d.; Ross and Solinger 2017). While the framework was developed by Black women in the United States, scholars have argued for its salience in the global context, where studies have documented that racialized, gendered, economic, and colonial structures of power have influenced population policy and, in turn, family planning programs (Senderowicz 2019; Kuumba 1999; Takeshita 2011; McCann 2017; Hartmann 1995). In the present study, the framework allows us to look beyond individual “bad actors” and examine larger structural forces that create an environment where people are unable to discontinue their LARC, echoing the conceptualization of contraceptive coercion as a structural phenomenon (Senderowicz 2019).

Instead of relating to individual people, providers, or programs, barriers to LARC removal may be perpetuated by the idea that contraceptive use—and particularly highly effective and long‐acting method use—is universally good for all people with the capacity for pregnancy, regardless of their contraceptive preferences (Senderowicz 2020). This belief is rooted in demographic and public health goals of eliminating unintended pregnancy to reduce the total fertility rate in the Global South, thereby framing individual contraceptive choices as the solution to big picture economic, health, and environmental challenges (Senderowicz and Valley 2023). Though the 1994 International Conference on Population and Development represented a shift in rhetoric toward voluntariness and choice (Senderowicz 2020; Nandagiri 2021), findings of the present study and many others reveal that some family planning programs do not center reproductive autonomy in their implementation approaches (Senderowicz and Kolenda 2022; Senderowicz 2019; Senderowicz et al. 2021; 2023; Britton et al. 2021). Scholars have repeatedly called for new measures of success in global family planning that do not view uptake and continuation of contraception as “good” and nonuse or discontinuation as “bad” (Holt et al. 2017, 2024, 2023; Speizer, Bremner, and Farid 2022; Senderowicz 2020). Measures that assess availability of a wide range of contraceptive methods, quality of contraceptive counseling, informed choice in contraceptive decision‐making, among others, are therefore vital. Several outcome measures that center people's desires around pregnancy and contraception have recently been developed in the United States and globally and can be implemented to evaluate family planning programs (Sudhinaraset et al. 2018; Holt et al. 2023; Dehlendorf et al. 2021; Bullington et al. 2025; Vincent et al. 2025; Senderowicz 2020; Harper et al. 2023). Additionally, enabling contraceptive discontinuation for those who desire to stop using their method is, as Senderowicz states, “an essential part of any rights‐based family planning approach” (Senderowicz and Kolenda 2022). Part of this work requires developing and implementing measures of access to LARC removal for the evaluation of programs that introduce or promote LARC. However, even though the PPIUD Study implemented such measures, access to LARC removal remained a barrier, perhaps due to the utilization of the monitoring dashboard that centered on IUD uptake and continuation as a marker of program success. Thus, in order to design truly rights‐based and person‐centered family planning programs, it is imperative that funders, program designers and implementers, and researchers agree that the fundamental goal of increasing access to contraception is not just to increase contraceptive prevalence, but rather to promote the human right of reproductive autonomy for all people across the globe.

Though research on the topic is limited, one way to potentially reduce barriers to IUD removal is through self‐removal. One study in the United States found that about half of the included participants were willing to try IUD self‐removal (Foster et al. 2014). Studies of social media and internet forums have revealed that people are removing their own implants and IUDs, and some do so after facing barriers to removal within the healthcare system (Amico et al. 2020; Broussard and Becker 2021). We echo Cartwright and colleagues' call for increased research on IUD self‐removal in LMICs (Cartwright et al. 2022). Additional clinical and acceptability research is needed to understand the safety and efficacy of self‐removal, as well as whether individuals in diverse settings are interested in self‐removal and whether providers are willing to educate patients about this option. While this is an exciting avenue for future research, IUD self‐removal also has inherent limitations. Some IUD users may not be interested in self‐removal, and those who are interested may face difficulties if, for example, their IUD strings are too short, leading to unsuccessful removals. Self‐removal is therefore likely not a comprehensive solution to barriers to LARC removal and the fundamental health systems problem of providers refusing to remove methods.

The present study represents an important step forward in prospectively and longitudinally assessing access to LARC removal following the implementation of family planning programs, highlighting the ways that programs can unintentionally inhibit reproductive autonomy through a focus on contraceptive uptake and continuation. Strengths include the utilization of multicountry longitudinal data, in which women were followed over nearly two years, and the innovative measures on barriers to LARC removal. The study also has some limitations. First, given that this is a secondary analysis, we relied on survey questions related to LARC removal that had already been implemented. While all participants who reported having their PPIUD deliberately removed were asked if they faced provider‐imposed barriers to removal (i.e., if a provider refused to remove their method), these participants were not asked about any other barriers to removal, such as cost or lack of supplies. We therefore posit that we underestimate the true proportion of participants who faced a barrier to PPIUD removal; we hypothesize that a higher proportion of participants who had their PPIUD removed faced a barrier to removal. There were likely additional participants who did not seek PPIUD removal, yet still desired to discontinue; given limitations to our survey, we were not able to capture this experience. Additionally, some participants who had their PPIUD deliberately removed did so during the two‐month follow‐up visit, during which they were given an examination to determine whether their PPIUD was in place and offered removal. Outside of this study setting, a higher proportion of participants may have faced barriers to removal. Further, we rely on participant self‐report of IUD expulsion, which may lead to measurement error. Additionally, attrition over the course of the study follow‐up may have led to selection bias; we addressed such bias by implementing inverse probability of observation weights.

## CONCLUSION

Our study finds that, following a provider‐focused intervention in PPIUDs, a substantial proportion of women who sought PPIUD removal faced barriers; most frequently, these were provider‐imposed. While some women eventually achieved removal, 9 percent of those who sought removal still had their PPIUD in use at the end of the study period. Our multicountry study therefore demonstrates persistent and prevalent barriers to IUD removal and highlights inconsistent counseling on removal with an imposable contraceptive method. These findings echo a large and growing body of evidence that reports pervasive barriers to LARC removal and calls for additional safeguards to ensure that all people who wish to discontinue their contraceptive method for any reason can do so.

Our findings demonstrate that, in the context of this PPIUD program, barriers to LARC removal may have been structural, rooted in the program's specific goals related to PPIUD use, which reflect larger demographic and public health goals that aim to promote contraceptive use over individual preferences. It is imperative to recall that developers of the IUD purposively made the method difficult for users to remove themselves (Takeshita 2011), and that provider‐imposed barriers to LARC removal have existed since the methods’ initial clinical trials (Hardee et al. 1994; Morsy 1995; Hardee, Balogh, and Villinskp 1997). The family planning field continues to grapple with a legacy shaped by systemic oppression, including racism, sexism, colonialism, eugenics, and reproductive coercion (Senderowicz and Valley 2023; Ginsburg and Rapp 1995; McCann 2017; Hartmann 1995; Ross and Solinger 2017). Acknowledging and incorporating this history into present day research is critical to contextualize findings and understand structural dynamics that constrain reproductive autonomy, including barriers to LARC removal.

## CONFLICT OF INTEREST STATEMENT

Data used in this analysis are publicly available in the Harvard Dataverse (https://dataverse.harvard.edu/dataverse/ppiud)

## ETHICAL APPROVAL

Ethical approval for this study was provided by the Ethics Review Committee at the Faculty of Medicine at the University of Colombo in Sri Lanka, the Nepal Health Research Council, and the National Institute for Medical Research in Tanzania. The Institutional Review Boards at Harvard University and the University of North Carolina at Chapel Hill deemed this study exempt.

## Supporting information



Supporting Information
