# Supplementary material for: Postpartum Intrauterine Device Removal and Access to Removal in the 18 Months Following an Intervention in Tanzania, Sri Lanka, and Nepal
Source: Stud Fam Plann. 2025 Oct 23;57(1):26–49. doi: 10.1111/sifp.70038 (PMC12996745; doi:10.1111/sifp.70038)
Supplement: Supplementary file 1 — Supporting Information [file SIFP-57-26-s001.docx]

**Table** A1 Intrauterine device use, expulsion, and removal in the 18 months following postpartum insertion^1^

|  | **Nepal** | **Sri Lanka** | **Tanzania** | **Total** |
| --- | --- | --- | --- | --- |
| Baseline n | 1,545 | 2,753 | 1,072 | 5,370 |
| **Two-month follow-up** | | | | |
| IUD in-use | 82% | 91% | 86% | 87% |
| Sought IUD removal services^2^ | - | - | - | - |
| IUD discontinued | 18% | 9% | 14% | 13% |
| Expelled^3^ | 5% | 3% | 2% | 4% |
| Deliberately removed | 12% | 6% | 12% | 9% |
| **Nine-month follow-up** |  |  |  |  |
| IUD in-use | 70% | 87% | 80% | 80% |
| Sought IUD removal services^2^ | 25% | 9% | 20% | 16% |
| IUD discontinued | 30% | 13% | 20% | 19% |
| Expelled | 6% | 5% | 3% | 5% |
| Deliberately removed | 24% | 8% | 17% | 14% |
| **Eighteen-month follow-up** |  |  |  |  |
| IUD in-use | 61% | 82% | 74% | 74% |
| Sought IUD removal services^2^ | 35% | 15% | 24% | 22% |
| IUD discontinued | 39% | 19% | 26% | 26% |
| Expelled | 7% | 5% | 5% | 6% |
| Deliberately removed | 32% | 13% | 22% | 20% |

^1^Due to use of inverse probability of observation weights and rounding, some observations may not add up as expected.

^2^Data on whether participants sought IUD removal services was not collected at two-month follow-up across all countries.

^3^Across all three countries, most participants attended the two-month follow-up visit in person (86% in Nepal, 59% in Sri Lanka, and 82% in Tanzania). Participants who attended in person and did not report that their PPIUD had been deliberately removed were given an examination, in which the status of their PPIUD was determined. Those who did not have a PPIUD present at two months but did not report that their PPIUD was deliberately removed were categorized as having their PPIUD expelled. For participants who attended two-month follow-up via phone, expulsion of PPIUD was determined by self-report.

TABLE A2 Barriers to intrauterine device removal and removal outcomes among participants who sought removal nine and eighteen months after postpartum insertion

|  | **Nepal** | **Sri Lanka** | **Tanzania** | **Total** |
| --- | --- | --- | --- | --- |
| **Nine-month follow-up** | | | | |
| N sought removal | 384 | 250 | 206 | 840 |
| Barriers to removal | | | | |
| Faced any barrier to removal^1^ | 18% | 16% | 18% | 17% |
| Faced a provider-imposed barrier to removal^2^ | 16% | 14% | 14% | 15% |
| IUD removal outcome |  |  |  |  |
| Removed | 95% | 91% | 86% | 92% |
| Not removed | 5% | 9% | 14% | 9% |
| **Eighteen-month follow-up** | | | | |
| N sought removal | 540 | 404 | 255 | 1,199 |
| Barriers to removal | | | | |
| Faced any barrier to removal^1^ | 24% | 25% | 25% | 25% |
| Faced a provider-imposed barrier to removal^2^ | 22% | 22% | 19% | 21% |
| IUD removal outcome |  |  |  |  |
| Removed | 92% | 88% | 91% | 91% |
| Not removed | 8% | 12% | 9% | 9% |

^1^Participants were categorized as facing any barrier to removal if they either: 1) reported that a provider had refused to remove their PPIUD, regardless of whether they were eventually able to achieve removal or 2) reported that they sought PPIUD removal but were not able to achieve removal.

^2^Participants were categorized as facing any barrier to removal if they answered “yes” to the question: “At any point, did a health service provider refuse to remove the PPIUD when you requested removal?”

|  | **Nepal** | | | | **Sri Lanka** | | | | **Tanzania** | | | |
| --- | --- | --- | --- | --- | --- | --- | --- | --- | --- | --- | --- | --- |
|  | IUD expelled | Did not seek removal | Sought removal and did not experience barriers | Sought removal and experienced barriers | IUD expelled | Did not seek removal | Sought removal and did not experience barriers | Sought removal and experienced barriers | IUD expelled | Did not seek removal | Sought removal and did not experience barriers | Sought removal and experienced barriers |
| **Total** | 6.9% | 58.1% | 26.5% | 8.5% | 5.2% | 80.1% | 11.0% | 3.6% | 4.5% | 71.6% | 18.0% | 5.9% |
| **Parity** (births) |  |  |  |  |  |  |  |  |  |  |  |  |
| 1 (ref) | 8.7% | 52.9% | 29.2% | 9.2% | 5.0% | 77.8% | 12.4% | 4.7% | 4.4% | 66.4% | 23.2% | 5.9% |
| 2 or more | 6.2% | 60.2% | 25.4% | 8.2% | 5.4% | 81.5% | 10.2% | 2.9% | 4.6% | 73.9% | 15.6% | 5.9% |
| **Age** (years) |  |  |  |  |  |  |  |  |  |  |  |  |
| 25 or older (ref) | 6.1% | 62.8% | 22.4% | 8.7% | 5.4% | 81.0% | 10.4% | 3.2% | 4.3% | 73.8% | 16.0% | 5.9% |
| Younger than 25 | 7.5% | 54.7% | 29.6% | 8.3% | 5.0% | 78.6% | 12.1% | 4.4% | 4.9% | 67.6% | 21.6% | 5.9% |
| **Education** |  |  |  |  |  |  |  |  |  |  |  |  |
| Completed secondary school (ref) | 5.0% | 62.9% | 22.6% | 9.6% | 4.6% | 79.4% | 12.1% | 3.9% | 2.3% | 71.3% | 18.6% | 7.9% |
| Less than secondary school | 8.3% | 54.7% | 29.3% | 7.7% | 7.5% | 82.7% | 7.2% | 2.6% | 5.9% | 71.8% | 17.6% | 4.7% |

TABLE A3 Probability that participants had their IUD expelled, did not seek IUD removal, sought IUD removal and did not experience barriers, and sought removal and experienced barriers by baseline characteristics (age, parity, education)

FIGURE A1 Flow diagram for four exclusive postpartum IUD outcomes
